# Supplementary material for: Host Cells Upregulate Phosphate Transporter PIT1 to Inhibit Ehrlichia chaffeensis Intracellular Growth
Source: Int J Mol Sci. 2024 Jul 19;25(14):7895. doi: 10.3390/ijms25147895 (PMC11276888; doi:10.3390/ijms25147895)
Supplement: Supplementary file 1 [file ijms-25-07895-s001.zip › Supplementary Materials -07-12.pdf]

Supplementary Figures

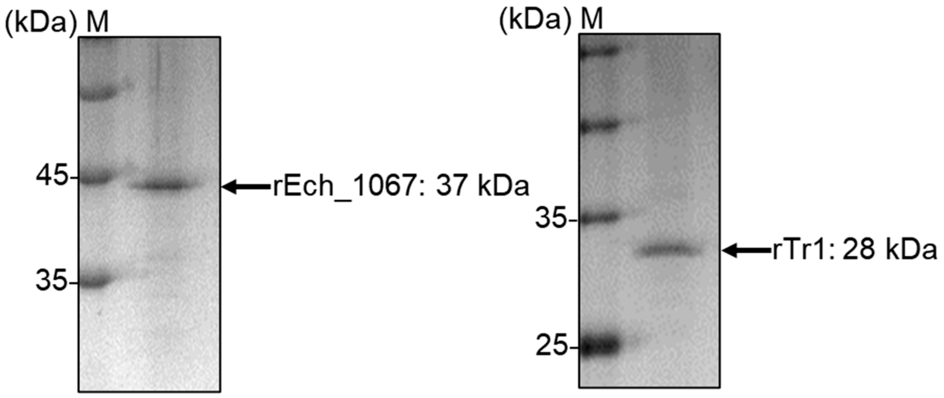

Figure S1. Purification of recombinant *E. chaffeensis* Ech_1067 and Tr1. *E. chaffeensis Ech_1067* without signal peptide (1-30 aa) or full-length *tr1* gene was cloned into the pET33b(+) vector to express rEch_1067 or rTr1. All proteins were purified with Ni-affinity chromatography. The purified proteins were subjected to SDS-PAGE, followed by Coomassie blue staining. M, protein marker. The purified proteins are indicated by arrows.

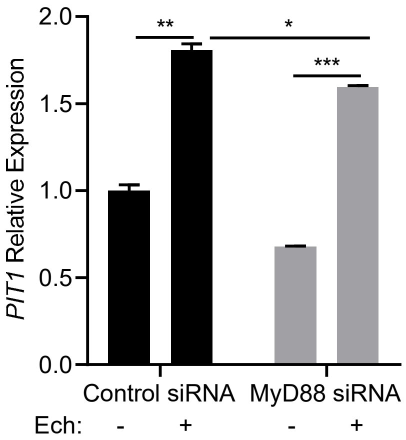

Figure S2. THP-1 cells upregulate the *PIT1* expression in a MyD88 dependent manner upon *E. chaffeensis* infection. THP-1 cells were transfected with siRNA targeting MyD88 or control siRNA for 24 h and then infected with isolated *E. chaffeensis*. At 24 h p.i., total RNA samples were extracted from the cells. The mRNA levels of *PIT1* were determined by qRT-PCR and normalized against those of human *GAPDH*. Data indicate means ± standard deviations ($n = 3$). The significant differences are represented by p-values determined with Student's t-test (***$p < 0.001$, **$p < 0.01$, *$p < 0.05$).

.

Supplementary Table

Supplementary Table S2. Bacterial strains, plasmids, primers, siRNA used in this study.

Strain/ Plasmid /Primer	Description	Source (Reference)
***E. coli* strains**		
BL21 (DE3)/Ech_1067	BL21 (DE3) expressing Ech_1067; Kan^r	This study
BL21 (DE3)/Tr1	BL21 (DE3) expressing Tr1; Kan^r	[1]
DH5α/pET-33b(+)-Ech_1067	DH5α harboring pET-33b(+)-Ech_1067	This study
**Plasmid**		
pET-33b(+)	Clone vector; Kan^r	Novagen
pET-33b(+)-rEch_1067	pET33b(+) harboring *Ech_1067* coding sequence; Kan^r	This study
pET-33b(+)-rTr1	pET33b(+) harboring *Tr1* coding sequence; Kan^r	[1]
**Primer**	**Sequence (5′→3′)**	**Function**
Ech_1067-F	GGGGGATCCGACAGCACCTCAAGCAGCAG	Protein expression
Ech_1067-R	GGGCTCGAGTTATGAAGTAGCAACTTTATCTG	Protein expression
16S rRNA-F (*E. chaffeensis*)	GGTGAGTAATGCGTAGGAATC	qRT-PCR
16S rRNA-R (*E. chaffeensis*)	GCTCATCTAATAGCGATAAATC	qRT-PCR
PIT1-F	GTTCGTGCATTCATCCTCCAT	qRT-PCR
PIT1-R	TGGTACCCCACAGAGGAAGTTT	qRT-PCR
GAPDH-F	ATCCCATCACCATCTTCCAG	qRT-PCR
GAPDH-R	CCTGCTTCACCACCTTCTTG	qRT-PCR
MyD88-F	GGCTGCTCTCAACATGCGA	qRT-PCR
MyD88-R	CTGTGTCCGCACGTTCAAGA	qRT-PCR
p65-F	CTGCAGTTTGATGATGAAGA	qRT-PCR
p65-R	TAGGCGAGTTATAGCCTCAG	qRT-PCR
**siRNA**		
p65	GATTGAGGAGAAACGTAAA	
MyD88	GCAAGGAATGTGACTTCCA	
**shRNA**		
PIT1 shRNA	GCCAATGATGTAGCAAATTCT	

The enzymes sites are indicated by the underline.
Kan^r, kanamycin resistance.

References

1. Duan, N.; Ma, X.; Cui, H.; Wang, Z.; Chai, Z.; Yan, J.; Li, X.; Feng, Y.; Cao, Y.; Jin, Y.; Bai, F.; Wu, W.; Rikihisa, Y.; Cheng, Z., Insights into the mechanism regulating the differential expression of the P28-OMP outer membrane proteins in obligatory intracellular pathogen *Ehrlichia chaffeensis*. *Emerging microbes & infections* **2021,** 10, (1), 461-471.
